# Lived experiences of shared decision-making in young adults prescribed antipsychotics: a qualitative interview study

**DOI:** 10.1007/s11096-026-02090-7

**Published:** 2026-02-24

**Authors:** Holly Grey, Laura Lindsey

**Affiliations:** School of Pharmacy, Newcastle University, Newcastle upon Tyne, UK

**Keywords:** Adverse effects, Antipsychotic agents, Decision making, Psychotic disorders, Shared

## Abstract

**Introduction:**

Serious mental illnesses, such as schizophrenia, schizoaffective or bipolar disorder, often starts in late teens or twenties. Adherence to antipsychotic treatment for serious mental illness is often poor. Involving service users in decisions about their treatment leads to improved clinical outcomes and adherence. Shared decision-making is bilateral information sharing between clinician and service user where decision-making is shared between both parties. This study aimed to evaluate the lived experiences of young adults on how healthcare professionals involve them in decisions about antipsychotic medication.

**Method:**

Fifteen semi-structured interviews were conducted online using Zoom. Participants were selected using purposive sampling via patient recruitment platform, support groups relevant to psychosis and social media. Those included in the study were diagnosed with schizophrenia, schizoaffective or bipolar disorder and treated with antipsychotics between the ages of 18–30. Data was coded inductively and thematic analysis was used to analyse the data.

**Results:**

Four themes were identified. These were living with antipsychotics, influence of family and friends, gaining autonomy and consequences of young adulthood. Findings highlighted the range of side effects from antipsychotic medication and their impact on young adults, and how the choices made by health care professionals influenced the quality of shared decision-making. Health care professionals’ decisions directly influence the quality of life for service users. Young adults with psychosis acknowledge the effectiveness of antipsychotics but see the side effects as significant obstacles in their lives.

**Conclusion:**

The study found that health care professionals provided limited acknowledgement and support for antipsychotic side effects, which significantly impacted the young adults’ lives. Young adults want to be fully informed of their medication and potential side effects but support from friends and family was a potential barrier. Changes in practice are needed including an adjustment in clinical language used by health care professionals, giving information to service users’ post-psychosis and ensuring health care professionals are trained in shared decision-making.

**Supplementary Information:**

The online version contains supplementary material available at 10.1007/s11096-026-02090-7.

## Impacts statements

- Young adults are the most likely age group to be diagnosed with serious mental illness and the least likely age group to adhere to their treatment.
- Shared decision-making helps to improve treatment outcomes.
- To improve shared decision-making for young adults health care professionals need to account for the impact of the serious mental illness and antipsychotic medication has on young adults.
- Young adults were often not involved in decisions about initiation of their medication so in any conversations about medications, focus on encouraging young adults to gain autonomy for their treatment may improve shared decision-making.

## Introduction

Globally, approximately 12% of the population lives with mental health conditions [1]. In the UK, one in six people experience a mental health problem each year, with annual increase of 8.9% in the number of adults with serious mental illness accessing community mental health services and year on year urgent crisis team referrals being 45% higher [2]. The median age of diagnosis across all mental illnesses is 18 years and nearly two thirds of diagnoses occur before the age of 25 [3].Serious mental illness includes bipolar disorder, borderline personality disorder, schizophrenia, post-traumatic stress disorder and major depressive issues. The life expectancy for people with serious mental illness is at least 15 years less than for those without [4]. Potential reasons for this are long term risks related to medications and higher suicide rates [5, 6].

For the acute and long term management of serious mental illness, antipsychotics are often the first line treatment. Antipsychotics are licensed for use in schizophrenia, and bipolar disorder, but can also be used to treat severe depression or anxiety. There is an increase in antipsychotic prescribing that has been noted globally [7]. For example, in Finland, a growing trend of prescribing antipsychotics to young people has been noted [8]. Antipsychotics are associated with a wide range of adverse effects which are common, numerous and vary in severity [9].

Side effects, are unwanted symptoms related to taking a medicine not providing the desired therapeutic effect. Antipsychotic side effects include extrapyramidal symptoms, sexual dysfunction, weight gain, diabetes, cardiovascular effects, hyperprolactinaemia, hypotension and neuroleptic malignant syndrome [9, 10]. A systematic review of adverse effects of antipsychotics identified sexual dysfunction as the most commonly occurring side effects in just over 50 percent of people on antipsychotics reporting these [11]. In youth, weight gain and sedation are the most prevalent side effects [12].. The adverse effects can be socially and economically damaging for the individual, with significant impact on relationships and employment [13]. The consequence of adverse effects is non-adherence and potentially reduced physical and mental well-being of the service user [10]. It is estimated that 74% of service users with chronic schizophrenia discontinue their medication within 18 months [14]. For bipolar disorder, adherence amongst younger individuals is lower than among the older [15].

Shared decision-making is considered central for recovery and delivery of mental health services [16]. National guidelines, such as the National Institute for Health and Care Excellence (NICE) in the UK advocates for the use of shared decision-making in serious mental illness [17]. Shared decision-making is bilateral sharing of information between clinician and service user for joint treatment decisions to be made, based on service user’s preferences and clinical evidence [18]. Currently in prescribing for serious mental illness, patient centred care including shared decision-making is not always achieved, with service users claiming a feeling of coercion, lack of information and stigma as the main barriers [19].

For healthcare professionals (HCPs) perceived lack of capacity and clinical urgency are the challenges for delivering person centred care in psychiatry [20]. Read et al. investigated service users’ experiences of antipsychotics, with 57.5% having a negative experience [21]. Service users report their preferences are not considered in the medication management, and they are not made aware about potential adverse effects prior to starting medication [22]. Shared decision-making has been linked to treatment optimisation, improved quality of life and improved clinician and service user relationship [23]. However, a cross-sectional study of psychiatric patients found that those with schizophrenia are less likely to be involved in clinical decisions [24].

Most service users feel excluded by HCPs and describe their approach as hostile and paternalistic [25]. For young adults, resistance from psychiatrist, limited consultation time and lack of self efficacy has been reported as potential barriers to shared decision-making [26]. The adverse effects are the primary influence in medication decisions, and past negative experiences are a significant factor in optimising antipsychotic medication [27]. There is a need for understanding and compassionate conversations with service users, so that alternative antipsychotics can be suggested to address antipsychotic side effects and their impact [25].

### Aim

This study aimed to evaluate the lived experiences of young adults on how healthcare professionals involve them in decisions about antipsychotic medication.

## Method

### Study design and recruitment

This study is reported according to the COREQ Checklist [28]. A qualitative design was used to gain an understanding of service users’ experiences with antipsychotics and how their relationship with HCPs shaped this. Semi-structured interviews were used as this allows in-depth conversation enabling the collection of rich and detailed data, critical for data analysis [29].

The study focused on adults with diagnoses of bipolar disorder, schizophrenia or schizoaffective disorder and treated with first or second generation antipsychotics between the ages of 18–30. All participants had to be aged 18 or over and be able to speak English, with access to an email and technology to participate in an online interview. There was no limit on geographical location.

Participants were recruited through purposive sampling using public participation platform Voice Global and social media (X and Facebook). The recruitment on Facebook was via *Schizoaffective Disorder & Mental Health Support* and *Schizophrenia, Schizoaffective, Bipolar, Psychosis, BPD, EUPD, DID, CPTSD Support* groups with permission from their administrators. When participants contacted the research team expressing interest, they were sent a participant information sheet and consent form. Participants provided written informed consent prior to the interview. No prior relationship was established with the participants before the start of the study.

### Data collection

The one-off interviews took place in October and November 2024. A topic guide was based on existing literature surrounding shared decision-making in serious mental illness (Supplementary material 1). Topics included: medication history, side effects, shared decision-making, preferences and values, barriers and facilitators, suggestions for improvement and long term perspectives. The topic guide was piloted with a service user, who fitted the age criteria and had lived experience with psychiatric medication. Changes to the topic guide following the pilot interview included asking questions about both in-person and virtual appointments and specifying general conversation or leaflets when talking about sharing clinical information.

All interviews were conducted by HG, a pharmacy student doing her final year research dissertation. Before the interviews, participants returned consent form electronically and verbal consent was confirmed at the start of each interview. Interviews were conducted via videoconferencing (Zoom version 6.2.3) and were audio recorded. Only interviewer and interviewee were present in all the interviews. No field notes were made during or after the interviews however, a debrief conversation was had with LL after interviews. Functionalities offered by Zoom, such as pass worded entry, were employed to ensure the use complied with UK privacy regulations. After the interview, each participant was emailed a debrief (Supplementary material 2), signposting to support websites and phone numbers tailored to their location. All interviews were audio recorded with consent, transcribed verbatim and anonymised.

### Data analysis

Inductive thematic data analysis was used to identify relevant themes in the data [30]. Audio recordings were transcribed manually. They were not returned to participants for comment. Analysis was conducted using NVivo version 15. Transcripts were re-read several times to ensure familiarity and identify initial points of interest. Each transcript was coded and analysed separately. Complete coding method was selected, to gather all information relevant to the research question then selectivity of codes was applied [31]. Coding was undertaken by HG and codes were discussed and developed with LL who checked the coding of all interviews for consistency. The researchers were in agreement that data sufficiency, the point at which no new concepts are identified and reoccurring similarity of responses was evident, was reached after 11 interviews but a further three interviews were carried out to confirm it [32].

An undergraduate female pharmacy student with little experience of serious mental illness conducted this study. Prior to the study, the student had studied research methods, consultation skills and the pharmaceutical care of serious mental illness. Additionally, interpretation of the data and development of themes was guided by the academic supervisor (LL) and conversations with placement supervisors in mental health setting.

Feedback on the findings was not requested from the participants.

### Ethics approval

This study received ethical approval from Newcastle University’s Faculty of Medical Sciences Ethics Committee on 22nd of October 2024 (Ref No. 2935/52133).

## Results

### Participant characteristics

Thirty people expressed interest in the study, and 19 gave consent. A total of 15 participants were interviewed; this included eight females and seven males. The interviews lasted between 28 and 70 min (mean 38 min). Participants were from the United Kingdom (UK), except for two females, one each from the United States of America and South Africa. One interview was excluded from the study due to suspicion of impostor participant [33]. Table 1 shows the participant demographics. Tables 2 and 3 outline the participants’ journey with antipsychotics. Antipsychotic side effects experienced by the participants are described in Table 4.

**Table 1:** Participant demographics

Characteristic	n (%)
*Gender*	
Female	8 (57)
Male	6 (43)
*Age range*	
18–21	1 (7)
22–25	8 (57)
26–29	3 (21)
30–33	1 (7)
44–47	1 (7)
*Country of residence*	
United Kingdom	12 (86)
United States	1 (7)
South Africa	1 (7)
*Interview duration (minutes)*	
28–30	2 (14)
31–40	7 (50)
41–50	1 (7)
51–60	3 (21)
61–70	1 (7)
*Camera on/off*	
Camera on	5 (36)
Camera off	9 (64)

**Table 2:** Current treatment

Participant	Current antipsychotics	Antipsychotic form	Length of time on antipsychotic
P1	1. Aripiprazole	1. Depot	1. 6 months
	2. Haloperidol	2. Oral	2. 2 weeks
P2	Clozapine	Tablet	4 years
P3	Unknown	Tablet	2 years
P4	Cariprazine	Tablet	1 year
P5	Olanzapine	Tablet	11 years
P6	Haloperidol	Tablet	1 year
P7	Haloperidol	Injection	
P8	Clozapine	Injection	7 years
P9	Chlorpromazine	Tablet	1 year
P10	Olanzapine	Injection	5 years
P11	Amisulpride	Tablet	2 years
P12	Risperidone	Tablet	3 weeks
P13	None	-	-
P14	Lumateperone	Tablet	1 year

**Table 3:** Diagnosis and previous treatment

Participant (age)	Diagnosis	Length of diagnosis	Previous antipsychotics
P1 (25)	1. Bipolar	1. 3 years	Risperidone
	2. Schizoaffective Disorder	2. 4 years	Quetiapine
			Aripiprazole
			Haloperidol
			Paliperidone
P2 (25)	Bipolar Disorder	5 years	None
P3 (28)	Bipolar Disorder	3 years	None
P4 (24)	1. Bipolar Disorder	1. 6 years	Aripiprazole
	2. Schizoaffective disorder	2. 4 months	Olanzapine
			Lurasidone
			Haloperidol
P5 (26)	Bipolar Disorder	11 years	None
P6 (27)	Bipolar Disorder	4 years	None
P7 (24)	Bipolar	7 years	None
	Disorder		
P8 (24)	Schizophrenia	7 years	None
P9 (25)	Bipolar Disorder	5 years	Sulpiride
P10 (23)	Schizophrenia	5 years	Clozapine
P11 (33)	Schizoaffective Disorder	7 years	Haloperidol
			Risperidone
P12 (22)	Bipolar Disorder	1 month	None
P13 (47)	1. Anxiety	28 years	Thioridazine
	2. Hallucinations/ paranoia		Aripiprazole
	3. C-PTSD		
	4. Bipolar Disorder		
	5. Severe Anxiety and major depression with Trauma		
P14 (21)	Bipolar Disorder	5 years	None

**Table 4:** Self reported antipsychotic side effects experienced by participants

Side effect	P1	P2	P3	P4	P5	P6	P7	P8	P9	P10	P11	P12	P13	P14
Dizziness			X			X	X		X	X	X			
Drowsiness								X	X					
Dry mouth		X	X										X	X
Extrapyramidal symptoms	X			X										
Fatigue		X		X	X		X				X	X		X
Headaches										X		X		
Heart problems	X										X			
Hyperprolactinemia	X													
Insomnia		X		X		X								
Nausea and vomiting						X	X	X				X		
Sexual dysfunction		X											X	
Vision		X									X	X		
Weight gain		X						X			X	X	X	X
Exacerbation of psychosis				X					X		X			

### Thematic analysis

Four themes identified were: living with antipsychotics, influence of family and friends, gaining autonomy and consequences of young adulthood. Each is presented in turn below.

#### Theme 1: Living with antipsychotics

Taking antipsychotic medication impacted the lives of all the participants. All participants experienced side effects with antipsychotics; the most prevalent side effects were fatigue (7/14), dizziness (6/14) and weight gain (5/14). Sexual dysfunction was mentioned by two participants. The side effects had a restrictive physical impact on participants’ lives. Participants also shared the negative impact the side effects had on their mental health, and the frustration behind how restrictive they were.

*“It deprives me from having my normal life.” P2*

*“I always want to be this vibrant person, always wanting to move about and doing what I want to do at a particular time. But you know I get restricted when I feel dizzy, I want to sleep” P3*

*“I feel I'm not free, you know. I can’t do things you know, I'm afraid.” P9*

When sharing about the antipsychotic side effects with their HCP participants felt they were not believed or listened to. Participants were not given the time to communicate with their HCP and discuss the impact of the side effects freely. For participants, the lack of bilateral communication influenced their acceptance and adherence of antipsychotics. Some said that their side effects were not taken seriously until something drastic happened.

*“By the time I threw up in the office and blood came out I think they got the message” P6*

*“It's devastated me that I'm not being listened to.” P13*

Most participants had experienced a dismissal of their side effects. Participants said their HCP described side effects as normal and they must deal with them rather than being offered with an alternative antipsychotic to address them. This stopped any implementation of the participant’s opinion into their own treatment plan and so forfeits person-centred care approach.

*“I think the vibe I get from my GP is that it's a small price to pay for sanity” P6*

*“Different people have different reactions to these things, different body systems and all of that. So I'm not the only one making this complaint” P10*

Some participants were given a potential solution by a HCP, for example a change in their medication, dose or even a medication used to combat the side effects. However, one participant expressed their dislike towards adding another medication into their regime, but this was ignored. Few were given non-pharmacological advice to help ease the side effects.

*“It seems like the doctor would rather put you on another medication and deal with the side effects.”. P4*

*“I was given some particular food to eat, some things to take and the amount of water intake I could have in a particular day.” P8*

Information given about the side effects was very poor, with many participants carrying out their own research. Five participants had received no information about side effects. Some had found information leaflets helpful in understanding the side effects. The reasons participants thought they were not given this information included their age, lack of clinical knowledge, capacity, and assumed paranoia.

*“So when I started having some of these side effects, I wasn’t too surprised. Because it is something I’ve read about, so it didn’t take me unaware.” P3*

*“I was given more like an information booklet and then I was also spoken with. I was talked to and so I was told to ask questions and all of that.” P10*

Many participants shared that when first starting medication they were often in psychosis, so were not involved in treatment decisions nor remember receiving information. It was their wider support network who often received the information. Despite coming out of psychosis and being on medication, information about treatment decisions were never respoken about, so participants were never involved in conversations.

*“I was put on them when I was with my mum, but I was quite deep in psychosis, so I don't ever remember that conversation.” P1*

*“And when you're in that kind of condition, the doctors don't really tell you much about the medicine.” P4*

For all participants, antipsychotics had been effective in managing their wellbeing. However they often felt they had no choice in taking it and were told it was critical to fit into society by HCPs rather than there be an open discussion.

*“The bottom line is, you have to take the medicine, and if you don’t, they'll give it to you by injection.” P4*

*“And you know if I want to go about my daily activities without stress, I just take it.” P9*

Nearly all participants had wanted to completely withdraw from their antipsychotic at some point, the adverse effects were the main reason given. Half of the participants felt comfortable discussing stopping their antipsychotic with their HCP. One participant shared that they met with resistance when mentioning topping.

*“[GP] actually tried to explain quite a lot of things. Why I needed it, how it’s going to*

*help me. I think those words he said to me gave me the boost to actually go for it” P3*

*“And he basically attacked me and said: Oh, no, you never do that. You can't do that.” P11*

#### Theme 2: Influence of family and friends

All participants had support from family members, friends or a partner as a young adult taking antipsychotics. Less than half of participants said they had an overall good experience regarding the support they had received. This support included not being forced into taking medication and given a choice in treatment decisions.

*“My mum would always say, [name], what do you want? Yes, my mum always seeks my opinion.” P12*

*“Well, it made me feel like I had people around me that actually did love me and want*

*to be there for me.” P14*

However other participants shared the negative experiences of involvement of family and shared decision-making, such as being excluded from the conversation, forced into taking medication and having information kept from them. Participants expressed their frustration of not being allowed to participate despite being a legal adult. Three participants specifically perceived their parents as a reason for their lack of shared decision-making.

*“I just wanted to be able to speak for myself, and, like, you know be in charge of my*

*own decisions for the healthcare.” P4*

*“I remember my mum being there, I was barely spoken to. I'm the patient and I'm a legal adult. But he did more of the talking with her. It didn't feel nice. I felt like a child, quite irritating.” P6*

*“There was no choice in the matter. It was mum and dad saying, “You going to do what the doctor says”. So my views didn't really come into it.” P13*

Some participants described that people close to them assumed they were untrustworthy and incapable of following their medication regime. They were constantly observed and felt the need to ask permission to participate in activities of normal life despite being an adult.

*“I wasn't trusted. It was with my mum I would actually have to go to her room every morning with a cup of water and just take it.” P6*

*“I feel insecure. I feel everything I do will be met with resistance. And I feel I need to take permission from someone for doing something.” P9*

Some participants described the positive influence of a HCP who went beyond required duties to support them. However, not all participants had such positive interactions with their HCPs. One participant recalled never having access to a psychiatrist. Many UK participants shared they had unlimited access to their lead HCP, with frequent face-to-face appointments and the access to call them.

*“Well, I would say maybe I was lucky to have an open and understanding healthcare provider, who saw past my age, and we had a great conversation.” P7*

*“I talk to him about anything. I feel the slightest [bipolar] hitting. I'm calling my doctor.*

*This is how I feel. This is how I am doing. What should I do? What should I not do?”*

*P12*

#### Theme 3: Gaining autonomy

Prior to starting antipsychotics, all participants said they knew very little about antipsychotics. All participants had done their own research and felt they now had a better understanding. With shared decision-making, a small number of participants defined it accurately. Most participants had a limited understanding of shared decision-making. Some believing it was when a decision was made by a guardian or simply giving consent.

*“The decision is it's not based on what the doctor alone said, [it] is based on how I feel P5*

*“I got to read from people who've got lived experiences, living with schizophrenia and*

*managing the condition with antipsychotic drugs.” P10*

Some participants felt they now had complete involvement in their medication, whilst others thought there had been improvements but still lacked full autonomy. For one participant, having access to their medications was complete involvement. Another described themselves as the barrier; in the beginning they were reluctant to engage in medication. Overall, participants recognised the positive influence of involvement in treatment decisions.

*“Because I was asked and I felt my opinion was heard, and I shared exactly how I felt at the point, so I feel I was totally involved.” P7*

*“Constant checking up constant decision making for me. Make sure I don't have to take giant decisions by myself.” P9*

All participants had barriers to their ability to advocate for themselves. Support from family and friends played an influential role in either improving ability or squashing it. However some participants showed a reliance on a caregiver in managing their condition, which attributed to a lack of understanding and acceptance of side effects.

*“I feel like there is a lens through which they already view people that need to take [antipsychotics] that they're incompetent of doing anything for themselves.” P6*

*“Well, at that time I felt like I couldn't really speak to them freely about what I was*

*experiencing, and I also feel like I wasn't really given the chance, like this kind of*

*welcoming space to kind of discuss what I could be experiencing, or what I was going*

*through” P14*

#### Theme 4: Consequences for young adulthood

Participants expressed the impact of the condition on their friendships and relationships, with some mentioning social exclusion and loss of friends. This was the most common stigma experienced by them. Others identified that appointments and medication regime made it hard for them to socialise with friends. Participants explained that as they transitioned into young adulthood, the condition, and specifically the side effects, impacted their sex life. Participants shared this with HCPs but felt nothing was done to manage them.

*“It sort of affected my sex life and it was quite embarrassing.” P1*

*“It really reduces the quality of my lifestyle, quality of my health, quality of my friends.*

*And that made me lose a lot of friends because they think I wasn’t normal anymore.”*

*P2*

When first diagnosed, all participants had experienced a sense of confusion and an initial rejection of the diagnosis after a lack of information received. For some, this led to psychosis and admittance to an inpatient setting. As a young adult, participants felt the diagnosis was not needed, and especially not one with antipsychotic treatment. A couple of participants had received antipsychotics before the age of 18, but with no diagnosis, only being defined as “weird” or “different”.

*At first, I didn't understand the gravity of it. I thought maybe I was ill for a while, but I’ll*

*get better, or I see in time. [Then] I still don't get better.” P8*

*“You can call it a mentality of a child, but I felt like I would never be diagnosed with any critical condition in my life.” P12*

At the beginning of their treatment, the medication being effective was the most important factor to all participants. Ten participants expressed that managing their condition was still the most important element to them, as they felt it gave them back the independence. Yet, for some, side effect management is now their main priority due to the limitations and anxieties in social situations.

*“I wanted the medication to work if it would be possible, without any side effect.” P8*

*“I was so relieved to be okay and safe.” P11*

## Discussion

### Statement of key findings

This study explored the lived experiences of young adults with serious mental illness and shared decision-making. A key finding of this study is the limited recognition and support from HCPs to young adults experiencing antipsychotic side effects. The side effects significantly affect their life as a young adult and thus influences their acceptance of the condition and its medication. Additionally, involvement from friends and family was a potential barrier for young adults to be active participants in the conversation about their medication regime. Young adults acknowledge the effectiveness of antipsychotics but see the side effects as significant obstacles in their lives that they face without the support of HCPs.

### Strengths and weaknesses

The diversity of participants’ diagnosis is a strength of this study, because it provides rich, detailed data of lived experience of serious mental illness in young adults. The study provides a wide range of detailed individual experiences, from young adults’ experience of antipsychotic medication, side effects and shared decision-making. As the participation criteria was taking antipsychotics between the ages of 18–30 but no restrictions were put on the participants current age, two of the participants were older than 30 thus their recall of the experiences may have been affected by the time lapsed since initiation. The study did not specifically focus on exploring differences between primary care and specialised mental health settings which could have given more nuanced understanding to shared decision making and the role of HCPs. Also, using Zoom for data collection may have led to selection bias due to digital exclusion. Inclusion of more international participants could potentially strengthen this study to make comparisons between differences in experiences across countries.

### Interpretation

The study found that young people experienced a lack of support from HCPs when they shared their experiences of side effects including dismissal; this finding is consistent with Helbling et al. (2006), who found that GPs perceive antipsychotic side effects as an acceptable compromise for their clinical efficacy [34]. Likewise, Begum et al. (2020) suggests HCPs underestimate the impact of taking antipsychotics on the quality of life of the service user [35]. Whilst there is little research on specific reactions of HCPs to side effects, research regarding the need to optimise antipsychotic medication use is plentiful, with adverse reactions being the limiting factor for successful treatment [23].

An association between severity of side effects and reaction of HCPs was clear in this study. Prior studies have also noted side effects being overlooked until they become severe, with considerable inconsistencies in monitoring [36]. Findings of this study highlighted the inconsistent monitoring and review of side effects, with responsibility placed on participants to inform HCPs. This leaves room for interpretation of required actions of HCPs [37].

The findings show the value of fully informing young adults of their medication in the form of either a conversation or physical resources. Prior studies have noted the importance of informing service users of potential side effects, to elicit a positive experience and improve clinician-service user relationship [21]. The reasons young adults in this study gave for not being invited to be involved reflect those of Sheperd et al. (2014), who found HCPs believed that those with serious mental illness are unable to understand clinical information and lack insight into their condition [20]. Not ensuring young adults are fully informed can facilitate distrust between service user and clinician, leading to a negative experience with limited shared decision-making.

Another finding of the study is that information can often be given to service users whilst in psychosis but never re-explained. This finding broadly supports the work of other studies linking service users experiencing psychosis with limited understanding [38, 39]. However, not ensuring information is shared afterwards, has not been identified in previous studies. Without service user involvement, treatment options are not made in line with service user’s preferences but often what the people supporting them think is important. Beck et al. (2021) criticises the Mental Health Act for often failing to ensure patient autonomy, and leading to care that is not person-centred [40]. Whilst this study does not specifically focus on individuals being detained, it does suggest that service users consent and values, especially when they are young adults, are not routinely clarified after antipsychotics have been initiated and the individual has not been able to consent at the time.

Another finding of this study was the detrimental effect side effects had on the lives of the young adults not only impacting them physically but also socially. There are similarities between attitudes expressed by participants in this study and those described by Kaushik et al. (2016) in a systematic review of stigma in children and adolescents with mental illness [41]. Similarly, Kaushik et al. (2016) notes the prevalence of stigma young adults face, with the emphasis on the misunderstanding from their peers, and even family [41]. This study found that service users currently navigate the challenges of young adulthood while the impact and reality of the antipsychotic side effects goes unacknowledged by their HCPs. This study, among others [10, 13], highlights the long lasting impact side effects have on the service user.

This study found that support from friends and family can act as a barrier for young adult involvement in antipsychotic medication management. This finding is contrary to previous studies, which have suggested that family support is influential in improving clinical outcomes as well as quality of life for the service user [42, 43]. Our findings show a potential disregard of service users, and loss of autonomy, which support can bring. This can lead the individual to become vulnerable to crisis and relapse, which can delay recovery in the long term.

### Implications for practice

There needs to be a recognition of potential power dynamics that have influenced young adults’ experiences with antipsychotics where the initiation of the medication has been often directed by prescribers and approved by family members. To improve shared decision-making, service users with lived experience of antipsychotics side effects should work collaboratively with HCPs to create online and physical resources to help young adults transition to having autonomy over their care. This will facilitate a more active role for young adults. Co-created online medicines resources for antipsychotics created for children and young people exist and could be used as model for developing these resources [44].

Most service users receive antipsychotics for the first time during psychosis, this often results in a lack of information being received and limited shared decision-making [38]. It could be beneficial for the young adults to have access to the HCP involved in the initiation of medication to discuss treatment decisions post-psychosis once they are stable. Nevertheless, considering barriers such as time constraints and availability of HCPs, involvement of the wider HCP team is essential. A potential solution is to involve pharmacists to improve quality of care and optimise antipsychotics [45]. However, further research is needed to optimise the monitoring and management of antipsychotic side effects for young adults.

Finally, training and education is needed for HCPs regarding how to best engage and support a young adult in their antipsychotic management. This should be done in line with current evidence and to inform service users, listen to their concerns, and to be aware of how the support from family and friends may influence this.

## Conclusion

This study explored young adults’ lived experiences antipsychotic medication management and shared decision making. Young adults found the side effects challenging but felt they received limited information and support for antipsychotic side effects from HCPs. Involvement from family and friends had negative impact on feeling involved in shared decision-making and reduced the sense of autonomy for young adults. To improve shared decision making and to facilitate better care for young adults on antipsychotic medication, ongoing support that is person centred is needed. This includes recognising that parental influence on decisions at the initiation of medication may have had impact on young person’s involvement and the importance of acknowledging the impact of side effects.

## Supplementary Information

Below is the link to the electronic supplementary material.

Supplementary file1 (DOCX 28 kb)

## Data Availability

The data is not available in a repository.
