# Supplementary material for: Lived experiences of shared decision-making in young adults prescribed antipsychotics: a qualitative interview study
Source: Int J Clin Pharm. 2026 Feb 24;48(3):949–59. doi: 10.1007/s11096-026-02090-7 (PMC13176059; doi:10.1007/s11096-026-02090-7)
Supplement: Supplementary file 1 — Supplementary file1 (DOCX 28 kb) [file 11096_2026_2090_MOESM1_ESM.docx]

# Supplementary material

**Topic Guide – Semi-Structured Interview**

**Shared Decision-Making and Awareness of Antipsychotic Side Effects in Young Adults**

**Introduction:**

- How are you today?

**Medication history:**

- Which antipsychotic do you take currently?
- What do you take your antipsychotic for?
- When were you diagnosed with said condition?
- How did it feel as a young adult to be diagnosed with this condition?
- How did you feel when you were first diagnosed with the condition?
- Do you have any other health conditions?
- What was the first antipsychotic you tried?
- How many different antipsychotics (forms and variations) have been implemented into your medication plan?
- Do you take any other medications for other mental health conditions?

**Side Effects:**

- Have you experienced any side effects because of your antipsychotic medication?
- Would you be able to share your experience of these side effects?
- As a young adult, how did these side effects impact your life?
- What kind of conversation did you have about side effects when you were started on the medication?
- If you weren’t, why do you think this was?
- What advice did you receive about managing the side effects?
- Were you started on any other medications to manage the side effects?
- Did you receive any advice about long term health risks ?
- Do you think you have any long term health problems as a result of your antipsychotic medication?
- If you were, what problems?
- If you weren’t given advice on either, why do you think this is?

**Shared Decision-Making:**

- What is your understanding of shared decision-making?
- Can you describe your experience with the healthcare professional that started your medication?
- Do you remember which HCPs were involved – doctors, pharmacists, nurses?
- Which, if any, healthcare professional made you feel most involved in the management of your condition?
- Other than healthcare professionals, did you have any other support from other individuals?
- How involved did you feel in your medication management at the start?
- How involved did you feel in your medication management now?
- Why did you feel involved/not involved?
- How involved did you want to be?
- Did you feel you could share your concerns with the healthcare professional and why?
- Why/why not?
- How comfortable did you feel comfortable discussing your medication in terms of side effects and changing medication?
- What was the reaction from the health care professionals?
- Were you included in these changes?
- Were you provided any information when your medication was changed?
- How often were you asked about your side effects?
- How often was your medication reviewed?
- Have you had any positive experiences?
- Have you ever wanted to stop your antipsychotic medication?
- If so, why?
- And if so, did you feel like you could speak to the healthcare professional about this?
- If so, in what ways did they help you?

**Preferences and Values:**

- What was most important to you with your medication?
- What did you know about antipsychotics before taking them?
- Did you have any preferences about your treatment?
- And what were they?
- What concerns did you have about antipsychotics?
- How well do you feel your values were respected in your treatment?
- What questions or concerns did family or friends (support) about your treatment?
- How well did you feel the healthcare professional considered your preferences whilst managing your medication?
- What information regarding how to access help/support if needed were you provided with?

**Long-term Perspectives:**

- What has been your overall experience with antipsychotics?
- Have they been effective in managing your health?
- How has your relationship changed with your healthcare professional?
- What is your experience now with antipsychotics and their side effects?
- What is your experience now with healthcare professionals?

**Barriers and Facilitators (Stigma):**

- Were there any barriers you faced in being involved in your medication management?
- What were these barriers?
- How did they make you feel?
- Would you say if you had a different medical condition that you may have been more involved?
- Would you say there is any stigma towards young adults with mental health conditions from a healthcare professionals’ perspective?
- What stigma is this?
- Have you ever experienced any stigma?
- How did it make you feel?
- How did you feel your young age affected how you were treated by healthcare professionals?
- How do you believe stigma stops people from receiving support?
- Why do you believe stigma stops people from receiving support?
- Has stigma stopped you from seeking support?

**Improvements:**

- Are there any suggestions you would like to make from your experience as a young adult with antipsychotic management, in particular their side effects?
- Is there anything you would say to a young adult facing the diagnosis of a mental health condition?

**Additional Comments:**

- Is there anything else you would like to share about your experience with shared decision-making and antipsychotic side effects?
- Do you have any questions?

**Closing Questions:**

- How are you feeling?
- Do you have any questions about the research?

**Debrief Document - UK**

Dear Participant,

Thank you for participating in an interview and taking part in my research study, it was a pleasure to interview you. Your time and attention are truly appreciated. I hope you found the interview enjoyable and helpful.

If you found any of the topics discussed in this interview to be distressing or upsetting or if you are in a crisis and need to talk to someone, here are some relevant mental health crisis helplines.

| **Helpline** | **Call** | **Text** | **Website/Email** | **Extra Info** |
| --- | --- | --- | --- | --- |
| **Mind** | 0300 102 1243 |  |  | 9am to 6pm Mon-Fri |
| **Samaritans** | 116 123  0808 164 0123 – Welsh |  | [jo@samaritans.org](mailto:jo@samaritans.org) | 24hrs, 365 days |
| **SANEline** | 0300 304 7000 |  |  | 4:30pm-10pm every day |
| **National Suicide Prevention Helpline UK** | 0800 689 5652 |  |  | 6pm-midnight every day |
| **Campaign Against Living Miserably (CALM)** | 0800 58 58 58 |  | [CALM webchat service](https://www.thecalmzone.net/help/webchat/). | 5pm-midnight every day |
| **Shout** |  | Text SHOUT to 85258 |  | 24/7 confidential text service |
| **Papyrus** | 0800 068 4141 | 07786 209 697 | [pat@papyrus-uk.org](mailto:pat@papyrus-uk.org) | If you’re under 35  24/7 |
| **Nightline** |  |  | [Nightline website](http://nightline.ac.uk/want-to-talk/) | If you’re a student – access to university night-time listening service – all operators are students. |
| **Switchboard** | 0300 330 0630 |  | [chris@switchboard.lgbt](mailto:chris@switchboard.lgbt) | Identify as gay, lesbian, bisexual or transgender.  10am-10pm |

**If you need urgent support, but your life is NOT at risk please contact one or more of the following:**

- Contact your care team if you use mental health services
- Call 111
- Telephone your GP for an urgent appointment
- Visit your local NHS walk-in/urgent care centre

**If you need urgent crisis support and you feel as though your life is at risk, please do not hesitate to call 999 or visit A&E.**

If you would like to be updated on the result of the study, please respond to this email and you will be contacted in due course. If you have any further queries or observations arising from this study, please feel free to write me an email.

Yours sincerely,

Holly Grey

Final Year MPharm Pharmacy Student

School Of Pharmacy, The Faculty of Medical Sciences, Newcastle University

King George VI Building, Newcastle upon Tyne, NE1 7RU
